# The Efficacy of Manual Therapy on Musculoskeletal Pain in Menopause: A Systematic Review

**DOI:** 10.3390/healthcare12181838

**Published:** 2024-09-13

**Authors:** João Espírito Santo, João Moita, Alexandre Nunes

**Affiliations:** 1 Escola Superior de Saúde Atlântica, 2730-036 Barcarena, Portugal; jpmoita@uatlantica.pt (J.M.); alexandre.nunes@ipiaget.pt (A.N.); 2 Portugal National Centre, Foundation COME Collaboration, 8125-196 Quarteira, Portugal; 3 Escola Superior de Educação de Beja, Instituto Politécnico de Beja, 7800-111 Beja, Portugal; 4 Escola Superior de Saúde Jean Piaget do Algarve, 8300-025 Silves, Portugal; 5 Insight: Centro de Investigação Piaget para o Desenvolvimento Humano e Ecológico, 1950-157 Lisboa, Portugal

**Keywords:** manual therapy, musculoskeletal pain, menopause

## Abstract

(1) Background: The aim of this review was to evaluate the effects of manual therapy for musculoskeletal pain in menopausal women. (2) Methods: A comprehensive search of manuscripts published from inception until 29 February 2024 was conducted on PubMed, EBSCO Information Services (EBSCOhost), Physiotherapy Evidence Database (Pedro), Web of Science, Scientific Electronic Library Online (SciELO), Cochrane Central Register of Controlled Clinical Trials, and Scopus using Medical Subject Headings (MeSH) and free terms. Randomized controlled trials (RCT) investigating the effects of manual therapy for musculoskeletal pain in postmenopausal women were included. Articles published in non-English or non-Portuguese languages, case reports, expert opinions, dissertations, conference papers, and gray literature were excluded. Studies were screened for population, musculoskeletal pain, intervention, and pain outcome by two independent reviewers using an ad hoc data extraction form. (3) Results: A total of 5 RCTs (4 with high risk of bias and 1 with low risk of bias) were included (total sample = 245), addressing thumb carpometacarpal osteoarthritis, thoracic kyphosis, chronic neck and backache, knee osteoarthritis, and sternocostal joint pain. The combined results of these studies showed improved musculoskeletal pain in menopausal women; duration of the follow-up was between 4 weeks to 6 months. Conclusions: The majority of studies included in this systematic review were effective in reducing musculoskeletal pain in menopausal women. These results come mainly from studies with a high risk of bias with small sample sizes, and the most representative follow-up period was short-term. Therefore, the results of this systematic review should be interpreted with caution.

## 1. Introduction

Menopause is a period of physiological change as women approach reproductive senescence. In clinical settings, healthcare professionals define menopausal states based on the menstrual history of women using the State of Reproductive Aging Workshop (STRAW + 10) criteria. The criteria for defining the premenopausal state include regular cycles with a stable cycle length of 21–35 days. To confirm that a woman is perimenopausal, her menstrual cycles must be irregular for over 1 year, or her last menstrual period must have occurred at least 2 but less than or equal to 11 months of amenorrhea. A postmenopausal state is characterized when there is no menstrual cycle for at least 12 months [1].

For many women it represents a transition period associated with deep changes at several levels, which may have a direct impact on women’s quality of life and their subsequent healthy aging [1,2,3,4,5,6,7,8,9,10]. The shift from the reproductive to the menopausal state causes several alterations due to the lowering of estrogen levels and gonadal hormone concentrations with a direct repercussion on the musculoskeletal (MSK) system, with MSK pain being one of the most frequently reported symptoms [11,12,13,14,15]. Approximately 50% of the adult population suffers from some sort of MSK pain. In fact, MSK pain is considered to be the leading cause of disability worldwide [16], affecting more than half of the female population at mid-age [17,18,19]. Epidemiological research focusing on the postmenopause stage reveals an alarming prevalence of MSK pain [20] that increases linearly from premenopause to perimenopause and then to postmenopause [21]. Women are approximately twice as likely to have joint pain and stiffness around the time of or after menopause than their premenopausal counterparts [15,22]. Related to this, diseases like osteoporosis are frequent in menopausal women, which affects nearly one out of three women over the age of 50 [23]. Also, osteoarthritis is the most common form of arthritis; the lifetime risk for women is 47% [24], and arthralgia is a menopausal symptom in at least 50% of women [25,26,27,28]. However, many epidemiological studies do not differentiate musculoskeletal pain from arthritis, so assessing the burden of arthralgia and arthritis in these populations is difficult [14].

Although clinical evidence is scarce, several non-pharmacological approaches are used to treat obstetric and gynecological pain-related symptoms [29,30]. Concerning manual therapy on menopause, several studies have shown that it is effective in relieving menopausal symptoms [31] and has proven to be one of the most widely used and preferred therapies for menopausal women [32,33].

Manual therapy is a form of physical treatment used by several healthcare professionals to treat MSK pain and disability through the use of mobilization and manipulation of the body’s neuro-musculoskeletal structures to improve mobility and function [34,35,36]. It has been pointed out as a highly beneficial therapeutic approach to MSK pain treatment, being more ecological and cost-effective, and with a lower risk of adverse events compared to pharmacotherapy [37,38,39].

There is evidence of manual therapy’s value in treating musculoskeletal conditions, such as low back pain [40,41,42], neck pain [43,44], knee osteoarthritis [45,46,47], shoulder primary adhesive capsulitis [48], and lateral elbow tendinopathy [49]. Nonetheless, to the best of our knowledge, no review studies were conducted on the efficacy of manual therapy in treating musculoskeletal pain in menopausal women. Therefore, the purpose of this systematic review is to verify the effects of manual therapy on the pain outcomes in menopausal women with musculoskeletal pain.

## 2. Methods

This systematic review protocol was registered at PROSPERO CRD42024504871 and was conducted according to the Preferred Reporting Items for Systematic Reviews and Meta-Analyses (PRISMA) 2020 guidelines [50]. The research question and the search strategy were defined according to the Population, Intervention, Control/Comparison, and Outcome (PICO) framework. The research question was as follows: “What is the evidence on the effect of manual therapy on menopause MSK pain symptoms?”

### 2.1. Literature Search Strategy

After defining the search terms, based on the Medical Subject Heading (MESH) descriptors, the following terms were used: menopause* OR perimenopause* OR postmenopause* AND musculoskeletal diseases OR pain OR arthrit* OR rhemat* OR osteoarth* OR tendInitis OR sciatica OR lumbago OR fibrositis* AND manual ther* OR osteopath* OR chiropract* OR myofascial release OR craniosacral therapy OR musculoskeletal Manipulations OR neuromuscular therapy OR trigger point. The search strategy used is shown in Appendix A.

Based on the pre-determined inclusion criteria, two reviewers (JES and AN) independently searched for relevant titles and abstracts using a comprehensive search strategy in the following electronic databases: PubMed, EBSCOhost, PEdro, Web of Science, SciELO, Cochrane Central Register of Controlled Clinical Trials, and Scopus, from their year of inception until 29 February 2024. After the identification of potential relevant references, full-text articles were obtained for review. If the two reviewers (JES, AN) were not in agreement on the inclusion of an article, a consensus meeting with a third party (JPM) was held. The search was limited to studies in the English and Portuguese languages.

### 2.2. Study Strategy

The reviewers JES and AN, using the predetermined search strategy, independently scanned for potentially relevant articles. After duplicate removal, the studies suitable for review through the inclusion and exclusion criteria were retrieved for in-depth analysis. A consensus meeting with a third party was held if the reviewers were not able to reach an agreement on the inclusion of a study. The software system for reference retrieval was EndNote^®^ (online version 21), used for further scrutiny and to remove duplicates.

The studies included in this systematic review were selected based on the following inclusion criteria: (1) manual therapy was defined as any hands-on technique or therapy applied by a health practitioner, which included moving joints in various and specific directions (joint mobilization and manipulation), stretching, passive range of motion movements of the affected body part, patient moving the body part against the therapist’s resistance (muscle energy techniques and proprioceptive neuromuscular facilitation), and soft tissue techniques (massage, fascial techniques) [41,51]; (2) only randomized controlled trials were included; (3) trails with a population featuring menopausal women with MSK pain; and (4) valid pain assessment outcomes through the use of validated scales such as the Visual Analogue Scale (VAS) or Numerical Pain Rating Scale (NPRS). All studies not meeting the inclusion criteria, expert opinions, dissertations, conference proceedings and abstracts, book chapters, and review papers were excluded. Although the review studies found were not included, their reference list was manually searched for additional relevant sources.

### 2.3. Data Extraction

The extraction of relevant data was conducted by the authors JES and AN, and in case of disagreement, a third author (JPM) was consulted to provide clarification. Because of the significant heterogeneity in the included studies’ design and methodological quality, meta-analysis was not performed and data were analyzed descriptively. After analysis, standardized summary of findings tables were created, concerning the following descriptive study characteristics: study first author/year and country; study design; population features; MSK condition; participants’ characteristics; group intervention; control intervention; outcome measurements; and results.

### 2.4. Methodological Quality Assessment

The methodological quality assessment of the included studies was evaluated independently by two researchers (JES and AN). The Cochrane collaboration risk-of-bias 2 (RoB 2) is the recommended tool for randomized controlled trials (RCT) [52]. The following five domains were assessed: (1) bias arising from the randomization process; (2) bias due to deviations from intended intervention; (3) bias due to missing outcome data; (4) bias in measurement of the outcome; and (5) bias related to selective outcome reporting. The Cochrane algorithm for each of the assessed domains and the overall judgments of risk of bias were rated as “high risk of bias”, “some concerns”, or “low risk of bias” [52]. The overall judgment was based on the following rule: low risk of bias—low risk of bias for all domains; some concerns—some concerns for at least one domain but no high risk of bias in any domain; high risk of bias—high risk of bias in at least one domain or some concerns in more than one domain [52]. The RoB2 tool is commonly used in systematic reviews to assess the risk of bias in RCTs [53].

The results were compared and irregularities were discussed until a final score was reached. A third reviewer (JM) was consulted in case of no agreement between the first two reviewers. The mentioned tool is the most commonly used in systematic review to assess the risk of bias in RCTs [54]. The strength of agreement between reviewers was determined through Cohen’s kappa [55]. This analysis was carried out with SPSS version 25.0 software (SPSS Inc., Chicago, IL, USA). The interpretation of k values was established using standards proposed by Landis and Koch [56]: 0 = poor agreement, 0.01–0.20 = slight agreement, 0.21–0.40 = fair agreement, 0.41–0.60 = moderate agreement, 0.61–0.80 = substantial agreement, and 0.81–1.00 = almost perfect or perfect agreement.

## 3. Results

### 3.1. Study Selection

**Figure 1:**
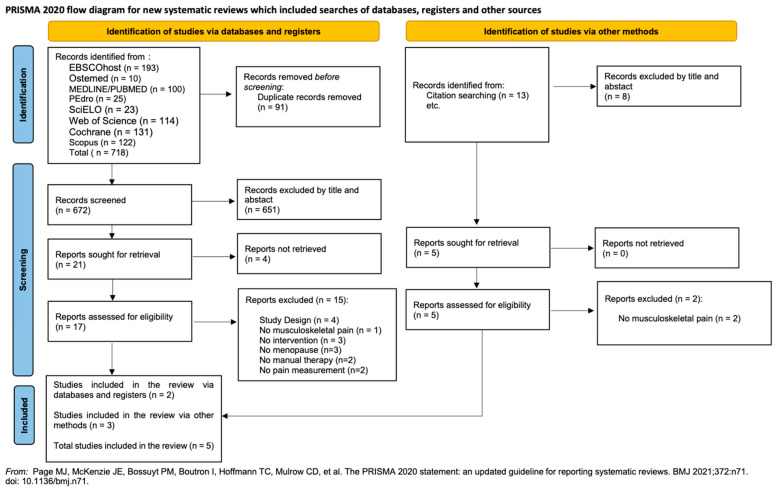
PRISMA flowchart [50].

Our search identified 731 potential studies. After duplicate removal, 677 titles and abstracts were screened for relevance, out of which 655 were excluded based on eligibility criteria and 22 were retrieved for in-depth full-text review. After full-text review, 17 studies were rejected: 3 did not address musculoskeletal pain; 3 had no intervention; 3 were on non-menopausal women; 2 did not address manual therapy; 2 did not included pain measurement; and 4 were of the wrong study type. A total of five articles met the inclusion criteria and were considered eligible for review. Figure 1 presents the flowchart concerning the process of searching for and selecting the studies included in this review.

### 3.2. Study Characteristics

The characteristics of the RCTs included in this systematic review are presented in Table 1. With regard to healthcare professions, 1 was from osteopathic medicine therapy [57], three were from physical therapy [58,59,60] and one was from Chinese traditional medicine [61]. The manual treatment techniques employed were proprioceptive neuromuscular facilitation [59], joint mobilization [58,60], joint manipulation [61], and Fox’s low-force osteopathic techniques [57].

### 3.3. Participants’ Characteristics and Musculoskeletal Conditions

The total sample size was 245 menopausal women with menopausal symptoms and MSK pain. The reported MSK conditions were thumb carpometacarpal osteoarthritis [59], thoracic kyphosis [58], chronic neck and backache [57], knee osteoarthritis [60], and sternocostal joint pain [61].

### 3.4. Main Results

In this systematic review, four studies reported significant improvement in pain between intervention and control groups [57,59,60,61], with follow-up durations ranging from 4 weeks to 6 months. One study showed no pain reduction but did report a decrease in thoracic kyphosis [58]. Proprioceptive neuromuscular facilitation (PNF) was effective in treating thumb carpometacarpal osteoarthritis, resulting in more significant pain reduction and disability level improvement than other interventions [59]. The osteopathic low-force techniques [57] and the manipulation of misaligned osteoporotic sternocostal joint pain [61] also showed significant pain reduction. Additionally, manual patellofemoral mobilization was found to effectively reduce pain in postmenopausal women with knee osteoarthritis [60].

### 3.5. Studies’ Quality Assessment

**Figure 2:**
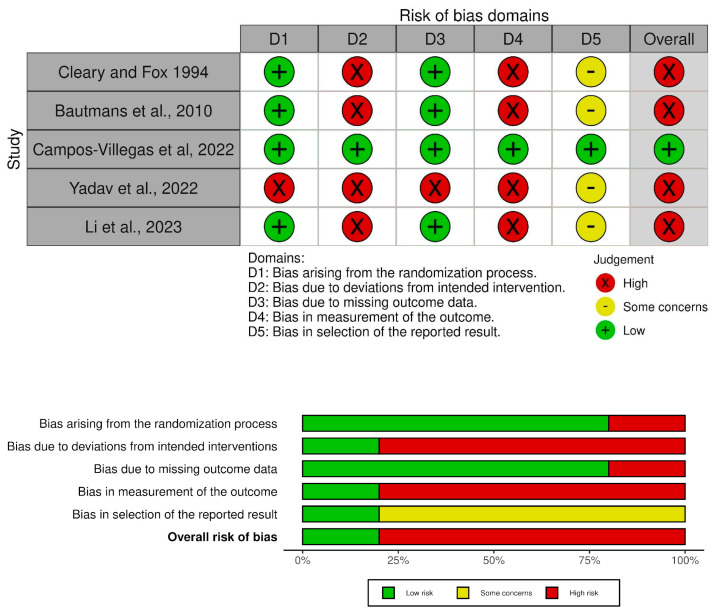
Risk-of-bias assessment of the included trials using the Cochrane Risk of Bias 2 for randomized controlled trials [57,58,59,60,61].

The methodology of the quality assessment based on RoB2 is summarized in Figure 2. In the RoB2 scale, only one RCT exhibited a low risk of bias [59], and four RCTs exhibited a high risk of bias [57,58,60,61]. Cohen’s kappa for the strength of agreement between reviewers concerning study quality was k = 0.75, demonstrating substantial agreement.

## 4. Discussion

This systematic review aimed to examine the efficacy of manual therapy on MSK pain in menopause women. Of the five studies included in this review, four reported pain reduction in the intervention group [57,59,60,61], whereas only one study revealed no differences between the interventions [58]. The aforementioned results correspond to one high-quality RCT study with a low risk of bias [59] and four studies classified as having a high risk of bias and/or low quality [57,58,60,61].

Pain is widely recognized as a multidimensional experience and is defined as such [62]. Evidence shows that menopause is a period of a woman’s life with various complaints that may affect her quality of life [63], among which MSK stands out [15,64,65]. In this sense, regarding the studies included in this systematic review, it is worth noting the high-quality RCT by Campos-Villegas et al. (2022) [59], which verified the efficacy of proprioceptive neuromuscular facilitation (PNF) in thumb carpometacarpal osteoarthritis. This was the first study on this condition in menopausal women mentioned by the authors. In a systematic review and meta-analysis [66] on the efficacy of manual therapy in the aforementioned musculoskeletal condition, moderate to high evidence of short-term effects was found. However, there was no clinically important difference in improving functional outcomes, such as strength or pressure pain threshold [66]. In addition, a recent systematic review and meta-analysis [67] on exercise-based interventions, in which most of the sample size were women (80%) with a mean age of 62 years, which included the study by Campos-Villegas et al. [59], found a statistically and clinically significant difference in the short-term reduction in pain intensity between proprioceptive exercise and exercise with manual therapy and orthoses [67]. According to the available knowledge, multimodal treatments that combine manual therapy and exercise are suggested to be the most effective for short-term improvements in pain reduction and functional capacity improvement, including in menopausal women with thumb carpometacarpal osteoarthritis.

Changes in estrogen levels have been associated with low back pain and osteoarthritis pain [68]. The more severe the menopausal symptoms, the greater the likelihood of associated suffering is [15,69]. Surprisingly, in this review, only two studies [57,58] included manual therapy to treat spinal musculoskeletal conditions, both with a high risk of bias. The conditions include thoracic kyphosis [58] and chronic neck or backache [57]. In different populations, manual therapy provides low-to-moderate evidence for reducing chronic non-specific neck pain [43], a significant effect in reducing acute neck pain, modest improvements in pain level concerning acute low back pain [70], and subacute low back pain [71]. It is worth mentioning that manual therapy is currently supported by many clinical practical guidelines for low back pain [40,41,42] and neck pain [72].

Osteoporosis is a highly prevalent condition in menopausal women and is a potential source of MSK pain [69]. Although osteoporosis is a contraindication for spinal manipulation [73], several studies recommend a gentle manual therapy approach, as in the study by Li et al. included in this review [61], which included manipulation of the misaligned osteoporotic sternocostal joint pain, with a statistically significant reduction in pain intensity in the intervention group. This was also seen in two cohort studies [74,75], in the cited systematic reviews [43,44,66], and in the intervention from Cleary and Fox [57] and Bautmans et al. [58], all of which have a high risk of bias. The latter two reported a reduction in pain levels [57] and in thoracic kyphosis curvature [58], a condition that can develop without the presence of vertebral fractures [76,77], considered a characteristic of senescence in humans [78,79]. In different populations, manual therapy reduces thoracic kyphosis in the short and medium term [80]. Hyperkyphosis has several implications, including increased falls, pain, and mortality, as well as a loss in quality of life and lung function (mechanical reduction in respiratory function, dyspnea, decreased vital capacity, and decreased forced expiratory volume) [81,82]. As a result, improving thoracic kyphosis is a clinically significant goal, and manual therapy is one approach to achieve it.

Similar to the findings of Yadav et al. [60], a systematic review showed that manual therapy in different populations provides short-term benefits in reducing pain [45] and is cost-effective [83] for the treatment of knee osteoarthritis.

Finally, in this review, two studies with a high risk of bias, in addition to reducing pain intensity, also reported diminished menopausal symptoms [57]. Systematic reviews have shown that manual therapy in menopausal women improves menopause symptoms such as insomnia and depression [84,85] and improves mental health [85].

### Limitations

There are some limitations to our study that should be considered. Most of the studies included in this systematic review had a high risk of bias and/or were low-quality studies. In addition, the total sample size for a systematic review was very low, and the most representative follow-up period was short-term. No pooled studies with the same musculoskeletal condition were conducted for a meta-analysis. This means that there was a wide variety of different conditions. Therefore, the results from this systematic review must be interpreted with caution.

Another limitation is the heterogeneity of the samples in the selected studies, which included postmenopausal, menopausal, or perimenopausal women with ages between 60 and 75 and different disorders. This diversity makes it difficult to compare the results. Therefore, any generalizations of these results should be limited to women with similar characteristics to those in our sample.

The few included studies in this review were without two studies that investigate a specific intervention for a common problem such as musculoskeletal pain in menopause; this is a substantial and frequently under-researched issue compared to the same theme in different populations. Thus, more research is needed to assess the effects of manual therapy in the treatment of musculoskeletal pain in menopause.

## 5. Conclusions

The findings of this review verified the efficacy of techniques for reducing musculoskeletal pain in menopausal women. The manual therapy techniques included were proprioceptive neuromuscular facilitation, osteopathic techniques, and joint mobilization and manipulation. They were compared with placebo manual techniques, strength training, or other types of exercise and waiting lists. Nevertheless, the results come mainly from studies with a high risk of bias, small sample sizes, and the most representative follow-up period was short-term. Therefore, the results of this systematic review should be interpreted with caution.

The number of studies included in this review highlights the need for additional research to investigate the efficacy of manual therapy in the treatment of musculoskeletal pain in menopause.

## Figures and Tables

**Table 1:** Characteristics of included studies.

First Author, Year, Country	Study Design	MSK Condition	Participants Characteristics	Group Intervention	Control Intervention	Outcome Measurements	Results
Campos-Villelas et al. (2022) [59], Spain	RCT	Postmenopausal with thumb carpometacarpal osteoarthritis.	N = 42 G1—n = 21 G2—n = 21 Mean age ± SD = G1 = 61.04 ± 6.11 G2 = 59.14 ± 8.05	G1—Proprioceptive neuro-facilitation—rhythmic stabilization alternate isometric contractions against resistance. A total of 3 sets, 15 rep, 5 s contraction, with 60 s rest between reps. Warm-up exercise.	G2—Strength training—Exercises were grip pinch, palmar pinch, key pinch, 3 sets, 10 reps, 5 s contraction with 60 s rest between reps. Warm-up exercise.	DASH VAS Kapandji test	Pain intensity baseline G1 = 5.91 ± 2.22 G2 = 5.26 ± 0.85 Follow-up (4 weeks) G1 = 2.37 ± 1.41 G2 = 4.94 ± 0.89 *p* < 0.001
Bautmans et al. (2010) [58], Belgic	RCT	Postmenopausal with thoracic kyphosis.	N = 48 G1—n = 29 G2—n = 19 Mean age ± SD = G1 = 75.2 ± 1.3 G2 = 77.6 ± 1.6	G1—Thoracic spine gentle spinal mobilizations with manual techniques—10–15 mobilizations. Exercises for postural correction—daily for 15–20 min. Taping—sets of elastic cure-tape once per week during three consecutive days.	G2—Waiting list for physical therapy.	VAS—back pain (0–100) Quality of life Mini Mental State Examination	Pain intensity baseline G1 = 33.6 ± 5.2 G2 = 29.5 ± 6.4 Follow-up (3 months) G1 = 33.9 ± 5.2 G2 = 31.3 ± 6.8 *p* = 0.920
Cleary and Fox (1994) [57], UK	RCT	Menopausal symptoms with chronic neck or backache.	N = 30 G1—n = 15 G2—n = 15 Mean age ± SD = NA	G1—Osteopathic low-force techniques against the restriction in the spine and pelvis, and cranial techniques. One treatment per week during 10 consecutive weeks.	G2—Placebo osteopathic low-force techniques in non-restricted joints and with the patient in neutral position, in the spine and pelvis, and cranial techniques. One treatment per week during 10 consecutive weeks.	VAS	Follow-up—15 weeks Neck pain *p* = 0.04 in favor G1 Back pain *p* = 0.06 in favor G1
Yadav et al. (2022) [60], India	RCT	Postmenopausal with knee osteoarthritis.	N = 45 G1—n = 15 G2—n = 15 G3—n = 15 Mean age ± SD = G1 = 52.33 ± 4.53 G2 = 52.40 ± 5.03 G3 = 52.33 ± 4.74	G1—Patello-femoral mobilization for 5 min comprising four to six mobilization series for 30–40 s for three sessions per week for 4 weeks.	G2—resistance training including strengthening of hamstrings and quadriceps muscle, for 40–45 min for three sessions per week for 4 weeks. G3—Control group received low-impact exercises with hot pack for 4 weeks.	VAS Knee range of motion flexion and extension WOMAC	Pain intensity baseline G1 = 8.06 ± 1.38 G2 = 7.93 ± 1.48 G3 = 7.66 ± 1.58 Follow-up—4 weeks G1 = 4.06 ± 1.57 (*p* < 0.05) G2 = 5.00 ± 1.55 (*p* > 0.05) G3 = 6.60 ± 1.80 (*p* > 0.05)
Li et al. (2023) [61], China	RCT	Perimenopausal with sternocostal joint pain.	N = 80 G1—n = 40 G2—n = 40 Mean age ± SD = G1 = 51.33 ± 2.60 G2 = 50.08 ± 3.19	G1—same treatment as the control group. Low-frequency pulsed electromagnetic fields at frequency 8 Hz and 12 Hz set at level 4 for 30 min, once a day for 10 days; kneading manipulation therapy at the misaligned sternocostal joint; the patient was asked to take a deep breath, and on the exhalation the therapist pressed downward until a snapping sound was heard.	G2—Control group—oral analgesics and calcium supplements. Aceclofenac 0.2 g orally once a day for 10 days and 600 mg calcium and 125 IU vitamine D3 orally 2 times/day for 6 months.	NRS Plasma calcium concentration Bone mineral density	Pain intensity baseline G1—7 (6.8) G2—7 (6.7) Follow-up—6 months G1—1 (0.2) *p* < 0.001 G2—2 (1.3)

DASH—disabilities of the arm, shoulder and hand; MSK—musculosekeletal; NDI—Neck Disability Index; NRS—Numerical Rating Scale; RCT—randomized controlled trial; VAS—visual analogue scale; WOMAC—Western Ontario and McMaster Osteoarthritis Index.

## Data Availability

Please contact the corresponding author to discuss the availability of the data and materials.

## Appendix A

**Table A1:** Search strategy used in PubMed, adapted from The Cochrane Collaboration Gross et al. [86] and Higgins and Green [87].

No.	Search Strategy
1	exp menopause/or exp perimenopause/or exp postmenopause/
2	Perimenopaus*.tw.
3	Postmenopaus*.tw.
4	Menopaus*.tw.
5	#1 OR #2 OR #3 OR #4
6	exp Musculoskeletal diseases/
7	(arthrit* OR rhemat* OR osteoarth* OR tendInitis OR sciatica OR lumbago OR fibrositis*).mp
8	#7 OR #8
9	Pain.tw
10	(pain OR painful OR analgesi*).mp
11	#9 OR #10
12	manual ther*.tw
13	osteopath*.tw
14	chiropract*.tw
15	myofascial release.tw
16	(cranio-sacral or craniosacral or cranio sacral therapy).mp
17	exp Musculoskeletal Manipulations/or deep tissue bodywork.tw
18	neuromuscular therapy.tw
19	trigger point.tw
20	#12 OR #13 OR #14 OR #15 OR #16 OR #17 OR #18 OR #19
21	Clinical Trial.tw
22	Randomized Controlled Trial.tw
23	random* allocate*.tw
24	Single Blind Procedure.tw
25	Double Blind Procedure.tw
26	Crossover Procedure.tw
27	Placebo.tw
28	prospective study.tw
29	cohort.tw
30	case study.tw
31	case report.tw.
32	#21 OR #22 OR #23 OR #24 OR #25 OR #26 OR #27 OR #28 OR #29 OR #30 OR #31
33	#5 AND #8 AND #11 AND #20 AND #32
